# Family caregiver roles and challenges in assisting patients with cancer treatment decision‐making: Analysis of data from a national survey

**DOI:** 10.1111/hex.13805

**Published:** 2023-07-02

**Authors:** James N. Dionne‐Odom, Erin E. Kent, Gabrielle B. Rocque, Andres Azuero, Erin R. Harrell, Shena Gazaway, Rhiannon D. Reed, Reed W. Bratches, Avery C. Bechthold, Kyungmi Lee, Frank Puga, Ellen Miller‐Sonet, Katherine A. Ornstein

**Affiliations:** ^1^ Department of Acute, Chronic and Continuing Care, School of Nursing University of Alabama at Birmingham Birmingham Alabama USA; ^2^ Division of Gerontology, Geriatrics, and Palliative Care, School of Medicine University of Alabama at Birmingham Birmingham Alabama USA; ^3^ Center for Palliative and Supportive Care University of Alabama at Birmingham Birmingham Alabama USA; ^4^ Department of Health Policy and Management, Gillings School of Global Public Health University of North Carolina at Chapel Hill Chapel Hill North Carolina USA; ^5^ Linebrger Comprehensive Cancer Center University of North Carolina at Chapel Hill Chapel Hill North Carolina USA; ^6^ Division of Hematology‐Oncology, School of Medicine University of Alabama at Birmingham Birmingham Alabama USA; ^7^ Department of Psychology University of Alabama Tuscaloosa Alabama USA; ^8^ Comprehensive Transplant Institute University of Alabama at Birmingham Birmingham Alabama USA; ^9^ CancerCare New York USA; ^10^ Center for Equity in Aging, School of Nursing Johns Hopkins University Baltimore Maryland USA

**Keywords:** cancer, decision‐making, family caregiving

## Abstract

**Background:**

We aimed to describe the roles and challenges of family caregivers involved in patients' cancer treatment decision‐making.

**Methods:**

Family caregiver‐reported data were analyzed from a national survey conducted in the United States by CancerCare® (2/2021–7/2021). Four select‐all‐that‐apply caregiver roles were explored: (1) observer (patient as primary decision‐maker); (2) primary decision‐maker; (3) shared decision‐maker with patient and (4) decision delegated to healthcare team. Roles were compared across five treatment decisions: where to get treatment, the treatment plan, second opinions, beginning treatment and stopping treatment. Ten challenges faced by caregivers (e.g., information, cost, treatment understanding) were then examined. *χ* ^2^ and regression analyses were used to assess associations between roles, decision areas, challenges and caregiver sociodemographics.

**Results:**

Of 2703 caregiver respondents, 87.6% reported involvement in patient decisions about cancer treatment, including 1661 who responded to a subsection further detailing their roles and challenges with specific treatment decisions. Amongst these 1661 caregivers, 22.2% reported an observing role, 21.3% a primary decision‐making role, 53.9% a shared decision‐making role and 18.1% a role delegating decisions to the healthcare team. Most caregivers (60.4%) faced ≥1 challenge, the most frequent being not knowing how treatments would affect the patient's physical condition (24.8%) and quality of life (23.2%). In multivariable models, being Hispanic/Latino/a was the strongest predictor of facing at least one challenge (*b* = −0.581, Wald = 10.69, *p* < .01).

**Conclusions:**

Most caregivers were involved in patients' cancer treatment decisions. The major challenge was not understanding how treatments would impact patients' physical health and quality of life. Challenges may be more commonly faced by Hispanic/Latino/a caregivers.

**Patient or Public Contribution:**

The CancerCare® survey was developed in partnership with caregiving services and research experts to describe the role of cancer family caregivers in patient decision‐making and assess their needs for support. All survey items were reviewed by a CancerCare advisory board that included five professional patient advocates and piloted by a CancerCare social worker and other staff who provide counselling to cancer caregivers.

## 1 INTRODUCTION

When patients receive a cancer diagnosis, a number of decisions about treatment have to be navigated. In most cases, patients consult with family members and close friends who know them well and are often greatly impacted by these decisions themselves.^1^, ^2^, ^3^ These family and friend caregivers assume a variety of decision support roles in cancer treatment decision‐making such as gathering information, providing emotional and psychosocial support, helping patients understand and process information, assisting with clarifying the patients' values and identifying decision points.^1^, ^2^, ^4^, ^5^

While identifying the various kinds of decision support roles (e.g., information gatherer, values and illness understanding discussant, option clarifier) that caregivers assume is becoming clearer, less is known proportions of *how* caregivers are partnering with their patients to make different treatment decisions. For example, it is unknown whether caregivers are more likely to serve as observers providing an opinion or instead serve as equal partners with patients in the treatment decision‐making process. Additionally, while qualitative reports have illuminated challenges faced by caregivers when assisting with decisions, such as having enough information or understanding costs,^1^, ^6^ little work has attempted to quantify proportions of individuals experiencing these challenges. Given the link between family involvement in decision‐making and patient outcomes such as satisfaction and treatment adherence,^6^, ^7^ understanding this type of systems‐level quantitative data is important to developing and testing broad strategies that enhance the support of families who partner with patients in their healthcare decisions.

Given this, we used data from a large national sample of cancer family caregivers in the United States to describe their involvement and role in patients' cancer treatment decision‐making and the challenges faced by family caregivers when assisting with these decisions. Furthermore, we explored associations between the sociodemographic characteristics of caregivers and patient clinical characteristics and the extent to which they encountered challenges to identify subpopulations who may be in most need of support.

## 2 MATERIALS AND METHODS

This was an analysis of data from a large national online US survey of 2703 family and friend caregivers of patients with cancer recruited through national consumer research panels from February to July 2021. The aim of the survey was to gain an understanding of cancer caregivers' needs and experiences in shared decision‐making.^8^, ^9^ The survey study was conducted by CancerCare®, a US nonprofit organization providing free, professional cancer support services. The survey was developed after focus groups with caregivers and social workers about the ways they support patients in treatment decision‐making and in partnership with experts in cancer family caregiving (including J. N. D.‐O. and E. M.‐S.). All survey items were reviewed by a CancerCare advisory board that included five professional patient advocates and piloted by a CancerCare social worker and other staff who provide counseling to cancer caregivers. The final survey included 63 items and is available in the Supporting Information: Appendix. Respondents were drawn from national market research panels in the United States vetted by PureSpecturm Inc. (a market research and insights platform), who self‐identified as a close friend or family member of an individual with cancer, 18 years of age or older and reported assisting with ‘health‐related decisions’. ‘Family caregiver’ was defined in the survey as an individual providing unpaid support in the past 12 months to a family member or friend who is close to them, who has cancer, and who did not have to live in the same home. The survey sample had approximately 25% coverage in each of the US Northeast, Midwest, Southeast, and Southwest/West regions. The study was deemed exempt by the University of Alabama at Birmingham Institutional Review Board after the survey data was deidentified and sent to the investigative team by Cancer*Care*.

### 2.1 Measures

#### 2.1.1 Demographic and clinical characteristics

Caregivers self‐reported data about their sociodemographics including age, gender, race, Hispanic/Latino ethnicity, education, geographic location, their relationship to the patient and the length of time they had been providing care. Caregiver respondents also reported the clinical characteristics of the patient, including the patient's cancer type and stage.

### 2.2 Items to measure treatment decision‐making, decision support roles and challenges

Determining caregiver respondents' involvement in cancer treatment decision‐making was done by evaluating an item set in the Cancer*Care* survey that asked them to check all of the different decision areas they had ever been involved in since providing support to their care recipient with cancer. Five of those items were queried specifically about decisions related to treatment. Those items included: ‘Deciding where to get treatment’, ‘Deciding whether to begin treatment’, ‘Deciding on the treatment plan (e.g., surgery, radiation, chemotherapy, immunotherapy, targeted therapy)’, ‘Getting a second opinion on the treatment plan’ and ‘Deciding whether or not to stop cancer treatment completely’. Subsequent to these items, the Cancer*Care* online survey had a ‘Decision Deep Dive’ section asking respondents to respond to further questions, which autopopulated on the online form, about their particular role and challenges faced within particular decision areas they ‘remembered the most clearly’.

Within the Deep Dive section of the Cancer*Care* survey, the respondent's decision support role was presented as four different types to which the respondent selected all the roles that represented the part they played in that particular cancer treatment decision area. The first was as an observer: ‘The person with cancer made the decision. I was an observer and played a supportive role’. The second was as the primary decision‐maker: ‘I made the decision. The person with cancer and other family and/or friends provided their input’. The third was as a shared decision‐maker: ‘The person with cancer and I made the decision together. We both agreed on the best choice’. The fourth was as a co‐delegator of the decision to the healthcare team: ‘The healthcare team made the decision. The person with cancer and I provided the input but the final decision was up to the healthcare team’.

Also, under the ‘Decision Deep Dive’ section, participants rated the extent to which they were faced with challenges concerning their involvement in a particular treatment decision area, using a set of 13 items representing possible difficulties. These items included: ‘Not everyone on the care team agreed’, ‘Some team members didn't agree with the doctor's recommendations’, ‘I didn't have enough information to make this decision’, ‘I didn't understand how the treatment would work’, ‘I didn't understand the out of pocket costs of treatments’, ‘I didn't know caregiver responsibilities for each of the treatment options’, ‘I didn't know how treatments would affect the person with cancer's physical condition’, ‘I didn't know how treatments would affect the person with cancer's quality of life’, ‘I didn't understand the treatment schedules’, ‘I didn't understand the risks and benefits of treatments’, and ‘I didn't know the wishes of the person with cancer’. Response options were: ‘Strongly agree’, ‘Somewhat agree’, ‘Neither agree or disagree’, ‘Somewhat disagree’ and ‘Strongly disagree’. Challenges in a caregiver's involvement were considered present for responses of ‘Strongly’ and ‘Somewhat’ agree.

### 2.3 Statistical approach

Descriptive statistics were used to characterize caregiver respondent‐reported sociodemographic and patient clinical characteristics. We assessed involvement in treatment decision‐making at both the level of the individual decision areas and in aggregate.

Using data from those respondents completing the ‘Decision Deep Dive’ section on one of the five cancer treatment decision‐making areas, we used cross‐tabulations and Pearson *χ* ^2^ tests to: (1) examine associations between reporting each of the caregiver roles in patient decision‐making and the five treatment decision areas; (2) examine associations between the five treatment decision‐making areas and reported challenges faced by caregivers in decision involvement and (3) assess associations between individual sociodemographic characteristics and experiencing one or more challenges when helping their care recipient with decisions.

Multinomial logistic regression was used to examine simultaneously the association between sociodemographic and patient clinical characteristics by reporting one or more challenges in helping patients with cancer treatment decisions. All analyses were conducted using IBM SPSS Statistics®, Version 25.

## 3 RESULTS

### 3.1 Demographics and clinical characteristics

There were a total of 2703 caregivers who responded to the survey, of whom 2367 (87.6%) reported involvement in at least one type of cancer treatment decision (Table 1). Of the 2367, 1661 completed the ‘Deep Dive’ portion of the survey focused on one of the cancer treatment decision areas where questions about roles and challenges were posed. The total sample (*N* = 2703), those who participated in at least one type of cancer treatment decision (*N* = 2367), and those who participated in at least one type of cancer treatment and completed the ‘Deep Dive’ questions (*N* = 1661) had similar proportions across all characteristics.

**Table 1:** Caregiver sociodemographic characteristics.

Characteristic	Total, *N* = 2703, %	Participated in cancer treatment decision‐making, *N* = 2367, %	Participated in cancer treatment decision‐making and responded to ‘Deep Dive’ questions, *N* = 1661, %
Caregiver age			
18–34	812 (30.0)	697 (29.5)	476 (28.7)
35–54	1307 (48.4)	1186 (50.1)	839 (50.5)
55 and older	578 (21.4)	481 (20.3)	343 (20.7)
Caregiver gender			
Male	1224 (45.3)	1103 (46.6)	793 (47.7)
Female	1434 (53.1)	1236 (52.2)	851 (51.2)
Transwoman/man or gender nonconforming	44 (1.6)	9 (0.5)	9 (0.5)
Caregiver race			
White	2106 (77.9)	1859 (78.5)	1322 (79.6)
African American/Black	342 (12.7)	286 (12.1)	183 (11.0)
Asian	154 (5.7)	137 (5.8)	95 (5.7)
Alaskan Native, American Indian, Native Hawaiian or Pacific Islander	33 (1.2)	23 (1.0)	18 (1.1)
Hispanic/Latino			
Yes	439 (16.2)	380 (16.1)	262 (15.8)
No	2256 (83.5)	1982 (83.7)	1395 (84.0)
Caregiver education			
Postgraduate degree	763 (28.2)	692 (29.2)	498 (30.0)
Some postgraduate	169 (6.3)	150 (6.3)	109 (6.6)
College graduate (4 years)	896 (33.1)	793 (33.5)	565 (34.0)
Vocational/technical school (2 years)	158 (5.8)	127 (5.4)	79 (4.8)
Some college	420 (15.5)	362 (15.3)	250 (15.1)
High school graduate or less	293 (10.8)	239 (10.1)	158 (9.5)
Caregiver total household income			
<$75,000	997 (36.9)	831 (35.1)	548 (33.0)
≥$75,000	1672 (61.9)	1510 (63.8)	1093 (65.8)
Location			
Urban	2253 (83.4)	1973 (83.4)	1394 (83.9)
Rural or small town	351 (13.0)	313 (13.2)	213 (12.8)
Caregiver–patient relationship (the patient is the caregiver's…)			
Parent	892 (33.0)	808 (34.1)	582 (35.0)
Friend	676 (25.0)	564 (23.8)	405 (24.4)
Spouse/partner	314 (11.6)	285 (12.0)	200 (12.0)
Sibling	162 (6.0)	139 (5.9)	89 (5.4)
Child	48 (1.8)	36 (1.5)	24 (1.4)
Extended family (e.g., aunt/uncle, grandparent, cousin)	587 (21.7)	514 (21.7)	348 (21.0)
Length of time providing care			
Up to 1 year	860 (31.8)	744 (31.4)	530 (31.9)
1–3 years	1160 (42.9)	1031 (43.6)	736 (44.3)
3–5 years	339 (12.5)	304 (12.8)	204 (12.3)
5 or more years	344 (12.7)	288 (12.2)	191 (11.5)
Patient's cancer type			
Solid tumour cancers^a^	2280 (84.4)	2017 (85.2)	1418 (85.4)
Haematologic cancers^b^	408 (15.1)	339 (14.3)	234 (14.1)
Patient's cancer stage			
In remission	566 (20.9)	453 (19.1)	312 (18.8)
1–2	970 (35.9)	855 (36.1)	629 (37.9)
3–4	1167 (43.2)	1059 (44.7)	720 (43.4)

^a^ Solid tumour cancer types: Bladder, brain, breast, colon/rectal, gynaecologic, head and neck, kidney, lung, melanoma, pancreatic, prostrate, thyroid.

^b^ Haematologic cancer types: Leukaemia, lymphoma, multiple myeloma.

Amongst the Deep Dive group (*n* = 1661), about half were between the ages of 35 and 54 (50.5%) and female (51.2%). Caregivers were White (79.6%), African American (11.0%) and Asian (5.7%). Over 16% (*n* = 439) were Hispanic/Latino/a. Most caregivers were the patient's child (35.0%), friend (24.4%) and spouse/partner (12.0%). The majority of patients had solid tumour cancer (85.4%) and slightly higher proportions of patients had stage 3–4 cancers (43.4%) than those with stage 1–2 cancers (37.9%) and those who were in remission (20.9%).

### 3.2 Roles of caregivers in patient decision‐making by decision area

Of the 1661 caregivers who were involved in cancer treatment decision‐making and completed the ‘Deep Dive’ portion of the survey, over half acted in the role of sharing these decisions with the patient (53.9%) (Table 2) and over 1‐in‐5 acted in the role of an observer (22.2%) and primary decision‐maker (21.3%). Overall, the most commonly reported decision area was where to get treatment (36.1%). Significant associations were observed between specific cancer treatment decision areas and reporting observer and primary decision‐maker roles (column *p*'s < .001). The largest proportion of caregivers in an observer role were those involved in decisions about the treatment plan (29.5%). The largest proportion of caregivers acting as a primary decision‐makers were those involved in decisions about where to get treatment (49.6%).

**Table 2:** Roles of caregivers in patient decision‐making by decision area.

Decision area	Total *N* = 1661	Observer supporter, patient primary decision maker, 22.2% (*N* = 369)	Caregivers as primary decision‐makers, 21.3% (*N* = 353)	Shared decision with the patient, 53.9% (*N* = 895)	The delegated decision to the healthcare team, 18.1% (*N* = 300)
Overall	Column % (*n*)	Column % (*n*)	Column % (*n*)	Column % (*n*)	Column % (*n*)
Deciding where to get treatment	36.1 (599)	24.4 (90)	49.6 (175)	36.1 (323)	33.3 (100)
Deciding on the treatment plan (e.g., surgery, radiation, chemotherapy, immunotherapy, targeted therapy)	27.0 (449)	29.5 (109)	21.0 (74)	28.7 (257)	31.7 (95)
Deciding to get a second opinion on the treatment plan	12.8 (213)	15.2 (56)	9.4 (33)	12.6 (113)	11.7 (35)
Deciding whether to begin treatment	17.6 (293)	19.8 (73)	14.7 (52)	17.7 (158)	18.7 (56)
Deciding whether or not to stop cancer treatment completely	6.4 (107)	11.1 (41)	5.4 (19)	4.9 (44)	4.7 (14)
Column *p* value^a^	n/a	<.001	<.001	.06	.20

*Note*: The sample sizes for individual decision areas were based on survey respondents selecting a decision they remembered clearly whereupon additional ‘Deep Dive’ section questions were asked about their role. Response options for the caregiver role (on the columns) were a ‘check all that apply’.

^a^ Pearson *χ* ^2^.

### 3.3 Challenges faced by family caregivers involved in treatment decisions

Out of 1661 caregivers, 60.4% (*n* = 1003) experienced at least one challenge when they were involved in their patient's treatment decision‐making. The most common challenges reported by caregivers were not knowing how treatment(s) would affect the person with cancer's physical condition (24.8%) and quality of life (23.2%) (Table 3). Associations between decision areas and specific challenges faced were found for all challenges (all column *p*'s < .001), with the highest proportion of challenges faced (for all challenges) observed for deciding on the treatment plan.

**Table 3:** Challenges faced by family caregivers involved in treatment decisions by treatment decision type^a^.

Decision area	Total *N* = 1661	Not everyone on the care team agreed, 13.7% (*N* = 228)	Some team members didn't agree with the doctor's recommendation, 13.5% (*N* = 224)	I didn't have enough info to make this decision, 11.8%, (*N* = 196)	I didn't understand how the treatment would work, 14.8% (*N* = 246)	I didn't understand the out‐of‐pocket costs of treatments, 17.3% (*N* = 287)	I didn't know caregiver responsibilities for each of the treatment options, 15.9% (*N* = 264)	I didn't know how treatment(s) would affect the person with cancer's physical condition, 24.8% (*N* = 412)	I didn't know how treatments would affect the person with cancer's quality of life, 23.2% (*N* = 386)	I didn't understand the treatment schedules, 10.4% (*N* = 172)	I didn't understand the risks and benefits of treatment, 13.4% (*N* = 222)
	Column %	Column % (*n*)	Column % (*n*)	Column % (*n*)	Column % (*n*)	Column % (*n*)	Column % (*n*)	Column % (*n*)	Column % (*n*)	Column % (*n*)	Column % (*n*)
Deciding where to get treatment	36.1	2.6 (6)	3.1 (7)	4.1 (8)	4.1 (10)	3.4 (10)	2.7 (7)	2.9 (12)	2.8 (11)	4.7 (8)	3.6 (8)
Deciding on the treatment plan (e.g., surgery, radiation, chemotherapy, immunotherapy, targeted therapy)	27.0	34.2 (78)	33.5 (75)	35.2 (69)	38.6 (95)	39.7 (114)	38. 3 (101)	43.7 (180)	43.3 (167)	38.9 (67)	36.5 (81)
Deciding to get a second opinion on the treatment plan	12.8	25.9 (59)	23.7 (53)	23.4 (46)	24.8 (61)	20.2 (58)	17.8 (47)	19.7 (81)	18.7 (72)	20.3 (35)	21.2 (47)
Deciding whether to begin treatment	17.6	23.7 (54)	24.1 (54)	24.5 (48)	32.5 (80)	26.4 (76)	31.4 (83)	26.2 (108)	26.7 (103)	36.0 (62)	28.8 (64)
Deciding whether or not to stop cancer treatment completely^b^	6.4	13.6 (31)	15.6 (35)	12.8 (25)	–	10.1 (29)	9.8 (26)	7.5 (31)	8.6 (33)	–	9.9 (22)
Column *p* value^c^	–	<.001	<.001	<.001	<.001	<.001	<.001	<.001	<.001	<.001	<.001

^a^ % of respondents for challenges areas who ‘somewhat’ or ‘strongly’ agreed with the statement.

^b^ Item stems altered to fit the context of stopping treatment (e.g., ‘Not everyone on the care team agreed about stopping treatment’, ‘I didn't understand the out‐of‐pocket costs of stopping treatment’).

^c^ Pearson *χ* ^2^.

Challenges reported by caregivers by demographic and patient cancer characteristics showed unadjusted differences in experiencing one or more challenges for younger caregivers, Hispanic/Latino/a caregivers, and caregivers with less formal educational attainment (Table 4). In multivariable models, Hispanic/Latino/a ethnicity was the strongest predictor of facing at least one challenge (*b* = −0.581, Wald = 10.69, *p* < .01).

**Table 4:** Challenges reported by caregivers by demographic and patient cancer characteristics.

Characteristic	Caregivers reporting ≥ 1 challenge(s) when helping patients with cancer treatment decisions, *n* (%)	Bivariate association	
		*p* Value^a^	Cramer's V
Overall (*N* = 1630)	**1093 (67.1)**	n/a	n/a
Caregiver age			
18–34	344 (71.2)	**.03**	0.07
35–54	514 (65.0)		
55 and older	232 (65.0)		
Caregiver gender			
Male	481 (65.6)	.23	0.04
Female	589 (67.9)		
Transwoman/man or gender nonconforming	23 (79.3)		
Caregiver race			
White	835 (65.6)	.25	0.05
African American/Black	153 (72.5)		
Asian	59 (68.6)		
Alaskan Native, American Indian, Native Hawaiian or Pacific Islander	14 (70.0)		
Hispanic/Latino			
Yes	200 (77.2)	**<.001**	0.10
No	888 (65.1)		
Caregiver education			
Postgraduate degree	281 (63.0)	**.048**	0.08
Some postgraduate	71 (70.3)		
College graduate (4 years)	365 (66.0)		
Vocational/technical school (2 years)	69 (75.0)		
Some college	184 (67.4)		
High school graduate or less	122 (74.9)		
Caregiver's total household income			
<$75,000	409 (68.1)	.45	0.02
≥$75,000	670 (66.2)		
Location			
Urban	903 (67.0)	.28	0.03
Rural or small town	134 (63.2)		
Caregiver‐patient relationship (the patient is the caregiver's…)			
Parent	366 (67.0)	.69	0.04
Friend	276 (68.7)		
Spouse/partner	120 (67.8)		
Sibling	69 (71.1)		
Child	20 (64.5)		
Extended family (e.g., aunt/uncle, grandparent, cousin)	231 (63.8)		
Length of time providing care			
Up to 1 year	366 (69.7)	.30	0.05
1–3 years	464 (66.8)		
3–5 years	139 (65.6)		
5 or more years	124 (62.6)		
Cancer type			
Solid tumour	907 (66.5)	.23	0.03
Haematologic	180 (70.3)		
Cancer stage—solid tumour			
1–2	381 (63.8)	.11	0.04
3–4	469 (68.0)		
Cancer stage—haematologic cancers			
0–2	71 (67.0)	.45	0.05
3–4	72 (72.0)		

*Note*: Bold values are statistically significant at *p* < 0.05

^a^ Pearson *χ* ^2^.

## 4 DISCUSSION

Family caregivers assume a variety of roles when supporting patients faced with cancer treatment decisions. However, little has been reported to date that quantifies the roles family caregivers play and the challenges faced when assisting with these decisions. To address this gap, we analyzed data from a large national survey of cancer family caregivers and found that a very high proportion (87.6%) were involved in patients' cancer treatment decision‐making. This finding in addition to others^2^, ^5^, ^10^ challenges the dominant clinical and research paradigm that has been guided by the two‐actor paradigm of shared decision‐making that narrowly focuses on the clinician and the patient.^11^, ^12^, ^13^

Just over half of caregivers (53.9%) had a shared role in making cancer treatment decisions with patients. Hobbs et al.^4^ published similar rates of sharing decisions about cancer treatment with family caregivers, as reported by over 5200 patients with lung and colorectal cancer. In their study, 49.4% reported sharing decisions with families. In our study, the treatment decision with the lowest rate of shared decision‐making with families was about whether or not to stop cancer treatment completely. Further study is needed to understand why caregivers are less likely to be shared decision‐makers for these decisions. Possible reasons for families being less involved include the belief that stopping treatment is ‘giving up’ or increases patient symptom burden, such as pain. Families may also worry about signalling a loss of optimism on behalf of a loved one, which is counter to being a ‘good’ family member or friend.^14^ It might also be the case that some oncology clinicians and/or the patients themselves believe this treatment decision should be dictated solely by the patient's wishes.^15^

The results suggest that, while families are highly involved in patient treatment decision‐making, how they are involved can differ across different decisions. A number of factors may explain these differences, such as (but not limited to) differences in perceived stakes of the decision including the severity of the patient's illness condition, patient preferences, family and cultural values, the perceived impact of the decisions on the caregiver's health, patient‐caregiver discordance on decisions, unique challenges of the sociodemographic context (e.g., access to care, insurance) and the treatment decision‐making conversation practices and communication skills of clinicians.^3^, ^10^, ^13^ A study of the decision‐making roles of 281 caregivers of patients with stage IV solid tumour cancers in Singapore by Ozdemir et al.^10^ reported that caregivers were more likely to be involved in decision‐making if those decisions had a higher impact on the caregiver's finances, schedule and health. This underscores how caregiver roles may vary based on the perceived impact of patient treatment decisions on family members and their financial circumstances and health.

Most caregivers (60.4%) faced one or more challenges when assisting with decisions, the most frequent being not knowing how treatments would affect the patient's physical condition (24.8%) and quality of life (23.2%). These challenges were especially notable for caregivers involved in decisions about the treatment plan. Recent research has found that large proportions of cancer caregivers lack or have misunderstandings of their care recipient's prognosis, survival and curability of the disease.^16^ There are several reasons why caregivers may have difficulties knowing how treatments might affect patients' quality of life. One reason is that prognostic information may not be effectively communicated (or not communicated at all) by the clinical team. Another reason may be the discordance in information needs during visits such that patients want to know very little about the treatment's impact on their lives and thus caregivers are unable to gain this information.^14^ Finally, some families may desire to maintain an optimistic and hopeful outlook in the face of their care recipient's poor prognosis such that the reality of the patient's current and future condition is distorted.^15^

Being Hispanic/Latino/a was the strongest predictor of facing at least one decision‐making challenge. Decision‐making challenges may stem from cultural factors that shape healthcare experiences for Hispanics/Latinos/as impacted by cancer. For example, Latinas with higher acculturation have been found to value participating in decision‐making more than less acculturated Latinas.^17^ Furthermore, Hispanic/Latino culture values collective decision‐making with family members giving input on healthcare decisions.^18^, ^19^ Other studies have noted high involvement by Hispanic families in patient decision‐making, with many attributing it to a cultural preference towards high family involvement and reliance on family to assist with English translation.^20^, ^21^ However, a survey of 387 Hispanic patients with advanced cancer by Yennurajalingam et al.^21^ found that only 34% had a preference for sharing decisions with families. Hence, our findings should be considered within the larger range of preferences by Hispanic individuals reported in the literature.

Our findings suggest several implications for clinical care, specifically decision support, of patients with advanced cancer and their families as they face numerous treatment decisions over the arc of care. First, clinicians should adopt a mindset towards shared decision‐making that moves beyond the patient–physician two‐actor paradigm and includes families in the decision‐making process. Second, clinicians may expect patients and families to differ on how the family member is involved in decisions, which could vary depending on the type of decision being made. Consequently, clinicians should discern the specific partnership on a case‐by‐case basis and tailor their decision support accordingly. Finally, caregivers can face a number of challenges when trying to support patient treatment decision‐making, particularly understanding the patient's physical condition and quality of life. Studies have shown the benefits of prognostic disclosure discussions,^22^, ^23^ hence clinicians should seek to initiate and conduct conversations with patients and families about prognosis and the likely course of the cancer trajectory. A growing body of resources is available to facilitate training in these conversation skills.^24^, ^25^

### 4.1 Study limitations

There are several limitations to this study. First, our survey may overestimate the proportion of caregivers involved in treatment decision‐making as the survey asked for respondents who had in some way been involved in ‘health‐related decision‐making’. Second, the CancerCare survey was cross‐sectional, thus we are unable to evaluate changes in caregiver roles and challenges over time. Future work should include longitudinal follow‐up to ascertain how caregiving decision‐making roles may change over time as the patient's cancer trajectory progresses. Third, the use of market research panels likely caused a selection bias towards individuals with access to the internet. Further, the use of these panels also impedes the ability to calculate survey response rates. These issues lessen the generalizability of the findings. Fourth, the survey sample had demographic characteristics that differ from other large population assessments of family; for example, this sample had a higher proportion of adult child family caregivers and a lower proportion of spouse/partner caregivers compared to other nationally representative surveys.^26^ Further, the survey did not collect other key background data to characterize the caregiving sample, such as the number of hours per week providing care. These considerations should also be considered when interpreting the applicability of the results. Finally, we ascertained the decision‐making roles of family caregivers based on their self‐report and not patients. There may be discordance in how patients viewed the caregiver's role, including patient preferences for how they would have liked caregivers to have been involved.^27^, ^28^

## 5 CONCLUSIONS

Using data from a large national survey, we found that the majority of family caregivers were involved in patients’ cancer treatment decisions. The biggest challenge in supporting patients in their treatment decision‐making was having a lack of information about how treatments would impact the care recipient's physical health and quality of life. Challenges in supporting patients were especially pronounced amongst Hispanic/Latino/a caregivers. These results in consort with a growing body of work in this area should prompt the development and refinement of strategies for assessing and including families in cancer treatment decision‐making.

## AUTHOR CONTRIBUTIONS

**James N. Dionne‐Odom**: Conceptualization, methodology, writing—original draft, writing—review and editing; **Erin E. Kent**: Conceptualization, methodology, writing—review and editing; **Gabrielle B. Rocque**: Writing—review and editing; **Andres Azuero**: Conceptualization, methodology, writing—review and editing; **Erin R. Harrell**: Conceptualization, writing—review and editing; **Shena Gazaway**: Conceptualization, writing—review and editing; **Rhiannon D. Reed**: Writing—review and editing; **Reed W. Bratches**: Writing—review and editing; **Avery C. Bechthold**: Writing—review and editing; **Kyungmi Lee**: Writing—review and editing; **Frank Puga**: Writing—review and editing; **Ellen Miller‐Sonet**: Funding acquisition, supervision, data curation, writing—review and editing; **Katherine A. Ornstein**: Conceptualization, methodology, writing—review and editing.

## CONFLICT OF INTEREST STATEMENT

The authors declare no conflict of interest.

## Supporting information

Supporting information.

## Data Availability

The data that support the findings of this study are available from Ms. Ellen Miller‐Sonet on behalf of CancerCare® upon reasonable request.
