# Supplementary material for: Family caregiver roles and challenges in assisting patients with cancer treatment decision‐making: Analysis of data from a national survey
Source: Health Expect. 2023 Jul 2;26(5):1965–76. doi: 10.1111/hex.13805 (PMC10485321; doi:10.1111/hex.13805)
Supplement: Supplementary file 1 — Supporting information. [file HEX-26--s001.docx]

Questions:

1. Let's begin by making sure our survey is right for you.

[free response]

2. First, please select the state you live in from the dropdown menu below.

[screener]

[singleSelect - text]

[fixed]

*a. Alabama

*b. Alaska

*c. Arizona

*d. Arkansas

*e. California

*f. Colorado

*g. Connecticut

*h. Delaware

*i. District of Columbia

*j. Florida

*k. Georgia

*l. Hawaii

*m. Idaho

*n. Illinois

*o. Indiana

*p. Iowa

*q. Kansas

*r. Kentucky

*s. Louisiana

*t. Maine

*u. Maryland

*v. Massachusetts

*w. Michigan

*x. Minnesota

*y. Mississippi

*z. Missouri

*A. Montana

*B. Nebraska

*C. Nevada

*D. New Hampshire

*E. New Jersey

*F. New Mexico

*G. New York

*H. North Carolina

*I. North Dakota

*J. Ohio

*K. Oklahoma

*L. Oregon

*M. Pennsylvania

*N. Rhode Island

*O. South Carolina

*P. South Dakota

*Q. Tennessee

*R. Texas

*S. Utah

*T. Vermont

*U. Virginia

*V. Washington

*W. West Virginia

*X. Wisconsin

*Y. Wyoming

3. In the past 12 months, have you provided unpaid support to someone close to you who has cancer (not including non-melanoma skin cancer)? This person can be a family member or friend and <u>does not have to</u> live with you?

[screener]

[singleSelect - text]

[fixed]

*a. Yes

*b. No (STOP)

*c. Prefer not to say (STOP)

4. Are you still providing this support?

[screener]

[singleSelect - text]

[fixed]

*a. Yes

*b. No (STOP)

*c. Prefer not to say (STOP)

5. In which of the following areas, if any, have you <b> <u>ever</b> </u> been involved in making decisions regarding the patient you care for? Please read the list carefully and select all that apply.

[screener]

[multiSelect - text]

[fixed]

*a. Deciding whether or not to get biomarker or genetic testing

*b. Deciding where to get treatment

*c. Deciding whether to begin treatment

*d. Deciding on the treatment plan (e.g., surgery, radiation, chemo, immunotherapy, targeted therapy)

*e. Deciding to get a second opinion on the treatment plan

*f. Deciding whether or not to be in a clinical trial

*g. Determining what medications to take to treat the symptoms and side effects of treatment

*h. Deciding whether or not to stop cancer treatment completely

*i. Deciding whether or not to get alternative, non-traditional therapy (such as high dose vitamins and supplements, homeopathy, chelation)

*j. Deciding whether to switch to another doctor or cancer center

*k. Deciding if the patient should go to the emergency room due to cancer symptoms or side effects

*l. Deciding whether or not to get palliative care

*m. Deciding whether or not to get rehabilitation

*n. Deciding whether or not to have hospice care

*o. Prefer not to say (STOP)

*p. I have not been involved in making any of these types of decisions (STOP)

6. What is your age and what is the age of the person you care for with cancer?

[grid]

~~ Row Statements ~~

*a. Your Age

*b. Patient's Age

~~ Column Choices ~~

*a. Under 18

*b. 18 to 24

*c. 25 to 34

*d. 35 to 44

*e. 45 to 54

*f. 55 to 64

*g. 65 to 74

*h. 75 or older

*i. Prefer not to say

7. Approximately how long have you been providing cancer related support/care for this patient?

[screener]

[singleSelect - text]

[fixed]

*a. Less than 6 months (STOP)

*b. Between 6 months to less than 1 year

*c. 1 to less than 3 years

*d. 3 to less than 5 years

*e. 5 to less than 7 years

*f. 7 to less than 9 years

*g. 9 to less than 10 years

*h. 10 years or longer

*i. Prefer not to say (STOP)

8. Next, please complete the following demographic questions for both you and the person you care for. We only ask these questions for classification purposes. They allow us to look at the results by different kinds of caregivers and patients to see how they differ. Again, all of this information is kept strictly confidential.

[free response]

9. What is your current gender identity and the patient's?

[grid]

~~ Row Statements ~~

*a. Your Gender Identity

*b. Patient Gender Identity

~~ Column Choices ~~

*a. Male

*b. Female

*c. Trans male<br>/trans man

*d. Trans female<br>/trans woman

*e. Genderqueer<br>/gender non-conforming

*f. Prefer not to say

10. Are either you or the patient of <u><b>Hispanic, Latino or Spanish origin of any race</u></b>?

[grid]

~~ Row Statements ~~

*a. You

*b. Patient

~~ Column Choices ~~

*a. Yes

*b. No

*c. Prefer not to say

11. Please indicate your race below. Again, please answer for both you and the patient.

[grid]

~~ Row Statements ~~

*a. Your Race

*b. Patient's Race

~~ Column Choices ~~

*a. White

*b. Black/African American

*c. Asian

*d. Native Hawaiian or Pacific Islander

*e. Alaskan Native or American Indian

*f. Two or more races

*g. Prefer not to say

12. Thank you. We are very interested in your responses to this survey. Please click the continue button to proceed.

[free response]

13. In this next section, we would like to know more about <b> <u>the patient </b> </u>for whom you provide care/support for.

[free response]

14. Which of the following best describes where the patient lives?

[singleSelect - text]

[fixed]

*a. Home/Apartment with me

*b. Home /Apartment, not with me

*c. Nursing Home

*d. Assisted living facility

*e. None of the above

*f. Prefer not to say

15. Approximately, how long does it typically take for you to travel by car to where the patient lives? Your best estimate is fine.

[singleSelect - text]

[fixed]

*a. Less than 5 minutes

*b. 5 to 15 minutes

*c. 16 to 30 minutes

*d. 31 to 45 minutes

*e. 46 to 60 minutes

*f. Longer than an hour but less than 2 hours

*g. Longer than 2 hours

*h. Don't know or prefer not to say

16. What is your relationship to the patient? <br> <b> The patient is... </b>

[singleSelect - text]

[shuffle]

*a. My spouse/partner

*b. My mother/father

*c. My child

*d. My brother/sister

*e. Someone in my extended family (aunt/uncle/grandparent/cousin/etc.)

*f. A friend

*g. Other [pinned]

*h. Prefer not to say [pinned]

17. What type of cancer was the patient diagnosed with?

[singleSelect - text]

[fixed]

*a. Bladder

*b. Brain

*c. Breast

*d. Cervical

*e. Colon or Rectal

*f. Head and Neck

*g. Kidney

*h. Leukemia

*i. Lung

*j. Lymphoma

*k. Melanoma

*l. Multiple Myeloma

*m. Ovarian

*n. Pancreatic

*o. Prostate

*p. Thyroid

*q. Endometrial/Uterine

*r. Other

*s. Prefer not to say

18. Approximately how long ago was the patient <b> <u>first diagnosed</b> </u> with cancer? Your best estimate is fine.

[singleSelect - text]

[fixed]

*a. Within the last six months

*b. 7 to 12 months ago

*c. 13 to 18 months ago

*d. 19 to 24 months ago

*e. More than 2 years ago

*f. Prefer not to say

19. What stage is the patient's cancer?

[singleSelect - text]

[fixed]

*a. Stage 1 or 2, it has <b> <u> not </u> </b>spread to other parts of the body

*b. Stage 3, it <b> <u>has spread</b> </u> beyond the tumor but not to nearby organs

*c. Stage 4, it <b> <u>has spread</b> </u> (metastasized) to other parts of the body

*d. In remission

*e. Don't know

*f. Prefer not to say

20. What stage is the patient's cancer?

[singleSelect - text]

[fixed]

*a. Stage 0, 1 or 2, high lymphocyte counts, swollen lymph nodes, and/or spleen and/or liver

*b. Stage 3 or 4, high levels of white blood cells, likely anemia

*c. In remission

*d. Don't know

*e. Prefer not to say

21. How would you describe the patient's <b><u>current treatment status</b></u>?

[singleSelect - text]

[fixed]

*a. Watchful waiting

*b. They are in active treatment

*c. They completed active treatment and are on maintenance therapy

*d. They are no longer in treatment

*e. Don't know

*f. Other

*g. Prefer not to say

22. Which of the following types of treatment, if any, has the patient <b><u>experienced</b></u>? Please check all that apply.

[multiSelect - text]

[shuffle]

*a. Surgery

*b. Chemotherapy

*c. Radiation

*d. Immune or biologic therapy

*e. Alternative, non-traditional treatment (such as high dose vitamins, homeopathy, chelation)

*f. Complementary treatment (such as acupuncture, massage therapy)

*g. Other [pinned]

*h. I am not sure [pinned]

*i. Prefer not to answer [pinned]

23. In this next section, we would like to know more about <u><b>your experiences as a caregiver</u></b> for this patient.

[free response]

24. In addition to yourself, who else provides <b>unpaid</b> care/support for this patient?

[singleSelect - text]

[random-v]

*a. No one else provides unpaid care besides me

*b. 1 other person (in addition to me)

*c. 2 other people (in addition to me)

*d. 3 or more other people (in addition to me)

*e. Prefer not to answer [pinned]

25. On average, how often do you provide some form of cancer related support to this patient? This support could be emotional support or any other activity where you are helping the patient cope with their illness. Your best estimate is fine.

[singleSelect - text]

[random-v]

*a. Daily

*b. Several Times Per Week

*c. Once Per Week

*d. 2 to 3 Times Per Month

*e. Monthly

*f. Less Often Than Monthly

*g. Prefer not to answer [pinned]

26. And thinking about the<u><b> typical day </u></b>when you provide care/support, approximately how many hours do you spend providing support for the patient? Your best estimate is fine.

[singleSelect - text]

[random-v]

*a. Less than 1 hour

*b. 1 to 2 hours

*c. 3 to 4 hours

*d. 5 to 6 hours

*e. 7 to 10 hours

*f. More than 10 hours

*g. Prefer not to answer [pinned]

27. In your opinion, how often can the patient communicate effectively with their cancer doctors without the help of others?

[singleSelect - text]

[fixed]

*a. All of the time

*b. Most of the time

*c. Sometimes

*d. Not usually

*e. Never

*f. Prefer not to answer

28. And in your opinion, how often can the patient care for themselves (dress, bathe, prepare and eat food) without the help of a caregiver?

[singleSelect - text]

[fixed]

*a. All of the time

*b. Most of the time

*c. Sometimes

*d. Not usually

*e. Never

*f. Prefer not to answer

29. In this next section, we would like to ask you some more questions about some of the decision areas you noted you were involved in.

[free response]

30. Of the following decisions you indicated you were involved in, which one do you <b><u>remember most clearly</u></b>?

[singleSelect - text]

[fixed]

*a. Deciding whether or not to get biomarker or genetic testing

*b. Deciding where to get treatment

*c. Deciding whether to begin treatment

*d. Deciding on the treatment plan (e.g., surgery, radiation, chemo, immunotherapy, targeted therapy)

*e. Deciding to get a second opinion on the treatment plan

*f. Deciding whether or not to be in a clinical trial

*g. Determining what medications to take to treat the symptoms and side effects of treatment

*h. Deciding whether or not to stop cancer treatment completely

*i. Deciding whether or not to get alternative, non-traditional therapy (such as high dose vitamins and supplements, homeopathy, chelation)

*j. Deciding whether to switch to another doctor or cancer center

*k. Deciding if the patient should go to the emergency room due to cancer symptoms or side effects

*l. Deciding whether or not to get palliative care

*m. Deciding whether or not to get rehabilitation

*n. Deciding whether or not to have hospice care

31. Regarding <b>$[Decision]</b> please describe the situation and your involvement in as much detail as you can. Please try to be as specific as possible.

[free response]

32. Please describe the part <b>you</b> played in <b>$[Decision]</b>.

[multiSelect - text]

[shuffle]

*a. The patient made the decision. <u>I was an observer</u> and played a supportive role.

*b. <u>I made the decision.</u> The patient and other family and/or friends provided their input.

*c. <u>The patient and I made the decision together.</u> We both agreed on the best choice.

*d. The clinical team made the decision. <u>The patient and I provided our input</u> but left the final decision up to the doctors and nurses.

*e. Other [pinned]

*f. Prefer not to answer [pinned]

33. Who was involved in <B>$[Decision]</b>? Check all that apply.

[multiSelect - text]

[shuffle]

*a. The patient

*b. Other family/close friends

*c. Faith or spiritual counselors

*d. The doctor and/or other clinical members of the cancer care team

*e. Other medical professionals not on the cancer team

*f. Other [pinned]

*g. Prefer not to answer [pinned]

34. In total, how many people were part of this decision making group?

[singleSelect - text]

[fixed]

*a. 0

*b. 1-3

*c. 4-6

*d. 7-10

*e. 11-15

*f. 15+

*g. Prefer not to answer

35. To whom and/or where did you go for help or information regarding this decision? (check all that apply)

[multiSelect - text]

[shuffle]

*a. The patient's clinical cancer care team

*b. Friends or family

*c. Medical professionals (other than the patient's cancer care team)

*d. The internet

*e. Patient education given to us by the patient's care team

*f. Social Media

*g. Government agencies or organizations

*h. Non-profit organization for caregiving or cancer

*i. Never looked for help or information [pinned]

*j. Prefer not to answer [pinned]

36. Thinking about <b>$[Decision]</b>, how much do you agree or disagree with the following statements?

[grid]

~~ Row Statements ~~

*a. The information I found or was given was not helpful

*b. The right decision was made

*c. I regret the choice that was made

*d. I would make the same choice if I had to do it over again and the situation was the same

*e. The choice that was made was harmful to the patient

~~ Column Choices ~~

*a. Strongly agree

*b. Somewhat agree

*c. Somewhat disagree

*d. Strongly disagree

*e. Prefer not to answer

37. In your experience as a family member or close friend of a person with cancer, has a nurse, doctor or social worker asked <b>you</b> about what <b>you</b> needed to help <u>you</u> share in making decisions regarding this patient?

[singleSelect - text]

[fixed]

*a. Yes

*b. No

*c. Not sure

*d. Prefer not to answer

38. In your own words, please tell us what was challenging about making this decision. Please be as specific as possible when answering.

[free response]

39. Regarding <b>$[Decision]</b>, please indicate how much you agree or disagree with the statements listed below.

[grid]

~~ Row Statements ~~

*a. Not everyone on the care team agreed

*b. Some team members didn't agree with the doctor's recommendation

*c. I didn't have enough information to make this decision

*d. I didn't understand how each of the treatments would work

*e. I didn't understand the out of pocket costs of the treatments

*f. I didn't know caregiver responsibilities for each of the treatment options

*g. I didn't know how the treatment would affect the patient's physical condition

*h. I didn't know how the treatments would affect the patient's quality of life

*i. I didn't understand the treatment schedules

*j. I didn't understand the risks and benefits of each treatment

~~ Column Choices ~~

*a. Strongly agree

*b. Somewhat agree

*c. Neither agree or disagree

*d. Somewhat disagree

*e. Strongly disagree

*f. Prefer not to answer

40. Regarding <b>$[Decision]</b>, please indicate how much you agree or disagree with the statements listed below.

[grid]

~~ Row Statements ~~

*a. Not everyone on the care team agreed

*b. Some team members didn't agree with the doctor's recommendations

*c. I didn't have enough information to make this decision

*d. I didn't understand the out of pocket costs of the treatments or tests

*e. I didn't know what testing would involve

*f. I didn't know what the findings might mean for family members

*g. I didn't know what the findings might mean for treatment options

*h. I didn't understand the treatment or test schedules

~~ Column Choices ~~

*a. Strongly agree

*b. Somewhat agree

*c. Neither agree or disagree

*d. Somewhat disagree

*e. Strongly disagree

*f. Prefer not to answer

41. Regarding <b>$[Decision]</b>, please indicate how much you agree or disagree with the statements listed below.

[grid]

~~ Row Statements ~~

*a. Not everyone on the care team agreed

*b. Some team members didn't agree with the doctor's recommendations

*c. I didn't have enough information to make this decision

*d. I didn't understand the out of pocket costs of the treatments or tests

*e. I didn't know about the treatment schedules

*f. I didn't know how cancer treatment differs by doctor or cancer center

~~ Column Choices ~~

*a. Strongly agree

*b. Somewhat agree

*c. Neither agree or disagree

*d. Somewhat disagree

*e. Strongly disagree

*f. Prefer not to answer

42. Regarding <b>$[Decision]</b>, please indicate how much you agree or disagree with the statements listed below.

[grid]

~~ Row Statements ~~

*a. Not everyone on the care team agreed

*b. Some team members didn't agree with the doctor's recommendations

*c. I didn't have enough information to make this decision

*d. I didn't understand how each of the medications would work

*e. I didn't understand the out of pocket costs of the medications

*f. I didn't know caregiver responsibilities for each of the treatment options

*g. I didn't know how the medication options would affect the patients physical condition

*h. I didn't know how the medication options would impact the patient's quality of life

*i. I didn't understand the medication doses and schedules

*j. I didn't understand the risks and benefits of each medication

~~ Column Choices ~~

*a. Strongly agree

*b. Somewhat agree

*c. Neither agree or disagree

*d. Somewhat disagree

*e. Strongly disagree

*f. Prefer not to answer

43. Regarding <b>$[Decision]</b>, please indicate how much you agree or disagree with the statements listed below.

[grid]

~~ Row Statements ~~

*a. Not everyone on the care team agreed

*b. Some team members didn't agree with the doctor's recommendations

*c. I didn't have enough information to make this decision

*d. I didn't understand the out of pocket costs of stopping treatment

*e. I didn't know caregiver responsibilities if treatment was stopped

*f. I didn't know how stopping treatment would affect the patient's physical condition

*g. I didn't know how stopping treatment would impact the patient's quality of life

*h. I didn't understand the risks and benefits of stopping treatment

~~ Column Choices ~~

*a. Strongly agree

*b. Somewhat agree

*c. Neither agree or disagree

*d. Somewhat disagree

*e. Strongly disagree

*f. Prefer not to answer

44. Regarding <b>$[Decision]</b>, please indicate how much you agree or disagree with the statements listed below.

[grid]

~~ Row Statements ~~

*a. Not everyone on the care team agreed

*b. Some team members didn't agree with the doctor's recommendations

*c. I didn't have enough information to make this decision

*d. I didn't understand if the patient really needed to go to the emergency room

*e. I didn't understand the out of pocket costs of going to the emergency room

*f. I didn't know how going to the emergency room would affect the patient's physical condition

*g. I didn't know how going to the emergency room would impact the patient's quality of life

*h. I didn't know how long the emergency room visit would take

*i. I didn't understand the risks and benefits of going to the emergency room

~~ Column Choices ~~

*a. Strongly agree

*b. Somewhat agree

*c. Neither agree or disagree

*d. Somewhat disagree

*e. Strongly disagree

*f. Prefer not to answer

45. When you were participating in <b>$[Decision]</b>, how important to <b>you</b> were these factors?

[grid]

~~ Row Statements ~~

*a. The patient's quality of life

*b. The impact of the choice on the patient's finances

*c. The patient's religious/spiritual beliefs

*d. The patient being able to continue working

*e. The patient's ability to take care of others (e.g., children)

*f. The patient's physical well-being

*g. The impact of the decision on the patient's being able to be at special events (e.g., weddings, births, travel plans)

*h. Changes in the patient's appearance such as hair loss or weight gain/loss

*i. The patient being able to function independently in activities of daily living (e.g., feeding, bathing,getting dressed)

*j. The impact of the decision on the patient's emotional well-being

*k. How long the patient is likely to live

*l. The opinions and feelings of other family members and friends

*m. The opinions of the patient's oncologist and healthcare team

*n. The consequences of this decision on my own day to day life

*o. My own religious and/or spiritual beliefs

~~ Column Choices ~~

*a. Very Unimportant

*b. Unimportant

*c. Important

*d. Very Important

*e. Not Applicable

*f. Prefer not to answer

46. Have you and the patient discussed any particular wishes the patient has about the care they would want to receive if they were dying?

[singleSelect - text]

[fixed]

*a. Yes

*b. No

*c. Not sure

*d. Prefer not to answer

47. How much does each statement describe how you feel about providing care for this close friend or family member with cancer?

[grid]

~~ Row Statements ~~

*a. I feel completely overwhelmed

*b. I feel useful and needed

*c. Caring for this person gives my life purpose and a sense of meaning

*d. I would not have chosen the situation I'm in, but I get satisfaction from providing care

*e. My family doesn't give me enough help

*f. The Lord won't give you more than you can handle

*g. I feel trapped by this person's illness

*h. In general, I feel competent as a caregiver

*i. Since becoming a caregiver I have become more aware of my inner strengths

*j. Since becoming a caregiver I have become more self-confident

*k. Since becoming a caregiver I have grown as a person

*l. Since becoming a caregiver I have learned to do things I didn't do before

*m. I trust myself to make the best decisions in caring for this person

*n. I am very satisfied with the care the doctors/clinical team provide

*o. I believe the doctors/clinical team value my input about treatment

~~ Column Choices ~~

*a. Does not <br>describe at all

*b. Describes<br>somewhat

*c. Describes<br>quite a bit

*d. Describes<br>completely

*e. Prefer not to answer

48. Regarding providing care for this close friend or family member with cancer, how often do/did you feel?

[grid]

~~ Row Statements ~~

*a. Adequately informed about the person's illness.

*b. Bothered that other family members have not shown interest in taking care of this person

*c. Communication in your family has improved

*d. Satisfied with the support you get from family

*e. You couldn't leave your relative alone

*f. Stressed between caring for this person and meeting other family or work responsibilities

*g. You have lost control of your life

*h. You have a loss of privacy and/or personal time

~~ Column Choices ~~

*a. Never

*b. Rarely

*c. Sometimes

*d. Often

*e. Nearly Always

*f. Prefer not to answer

49. Have you felt that the support you and the patient have received from the doctor or healthcare team for making cancer-related decisions has been negatively affected by any of the following:

[grid]

~~ Row Statements ~~

*a. Age

*b. Race

*c. Language

*d. Education level

*e. Health or disability

*f. Internet access

*g. Political affiliation

*h. Body weight

*i. Insurance type or lack of insurance

*j. Income level

*k. Religion

*l. Sexual orientation

*m. Gender/sex

~~ Column Choices ~~

*a. Has had a <br>negative effect

*b. Has NOT<br>had a negative effect

*c. Prefer not to answer

50. Please indicate how helpful or unhelpful the following services would be to you when you are involved in helping make treatment decisions for a close friend or family member

[grid]

~~ Row Statements ~~

*a. On-line group of caregivers in similar situations

*b. On-line group of patients with same health conditions as my friend/family member

*c. Free consultation with an oncology nurse

*d. Educational materials about cancer from a trusted source

*e. Free consultation with an oncology social worker

*f. Free consultation with an oncology doctor

*g. Videos about treatment decision making

*h. Worksheets that help guide decision making

*i. Role playing how to share cancer treatment decision making

~~ Column Choices ~~

*a. Not helpful

*b. A little helpful

*c. Very helpful

*d. Prefer not to answer

51. Over the <b><u>last 2 weeks </b></u>, how often have you been bothered by the following problems?

[grid]

~~ Row Statements ~~

*a. Feeling nervous, anxious, or on edge

*b. Not being able to stop or control worrying

*c. Little interest or pleasure in doing things

*d. Feeling down, depressed or hopeless

~~ Column Choices ~~

*a. Not At All

*b. Several Days

*c. More than half the days

*d. Nearly Every Day

*e. Prefer not to say

52. You are almost finished! Just a few more demographic questions about you and the patient.

[free response]

53. Please indicate your <b><u>marital status</b></u> and the marital status of the patient below.

[grid]

~~ Row Statements ~~

*a. You

*b. Patient

~~ Column Choices ~~

*a. Married

*b. Domestic partnership

*c. Single, living with a partner

*d. Single (including divorced, widowed or separated)

*e. Prefer not to say

54. Please indicate your <b><u>employment status</b></u> and the employment status of the patient below.

[grid]

~~ Row Statements ~~

*a. You

*b. Patient

~~ Column Choices ~~

*a. Working full time (30+ hours per week)

*b. Working part time (less than 30 hours per week)

*c. Retired

*d. Student

*e. Not employed, but looking for work

*f. Not employed, but not looking for work

*g. Prefer not to say

55. Please indicate the <b><u> highest level of education </b></u> you and the patient have completed.

[grid]

~~ Row Statements ~~

*a. Your Education

*b. Patient's Education

~~ Column Choices ~~

*a. Some High School or Less

*b. High School Graduate

*c. Vocational/Technical School (2 Year)

*d. Some College

*e. College Graduate (4 Year)

*f. Some Post-Graduate

*g. Post Graduate Degree

*h. Prefer not to say

56. And what type of <b><u>medical insurance</b></u> do you and the patient have, if any?

[grid]

~~ Row Statements ~~

*a. Your Medical Insurance

*b. Patient's Medical Insurance

~~ Column Choices ~~

*a. Medicare

*b. Medicaid

*c. Employer insurance

*d. Marketplace exchange (Affordable Care Act)

*e. Other

*f. No Insurance

*g. Prefer not to answer

57. Please indicate which of the following categories best describes your <b><u> total annual household</b></u> income before taxes. <br>

[singleSelect - text]

[fixed]

*a. $0

*b. $1 to $9,999

*c. $10,000 to $24,999

*d. $25,000 to 49,999

*e. $50,000 to 74,999

*f. $75,000 to 99,999

*g. $100,000 to 149,999

*h. $150 000 and greater

*i. Prefer not to answer

58. Please indicate which of the following categories best describes the patient's<b><u> total annual household income </u></b> before taxes.

[singleSelect - text]

[fixed]

*a. $0

*b. $1 to $9,999

*c. $10,000 to $24,999

*d. $25,000 to 49,999

*e. $50,000 to 74,999

*f. $75,000 to 99,999

*g. $100,000 to 149,999

*h. $150 000 and greater

*i. Prefer not to answer

59. Have you or the patient been diagnosed with COVID-19? Please select all that apply.

[multiSelect - text]

[fixed]

*a. I have

*b. The patient has

*c. No, neither of has been diagnosed

*d. Prefer not to answer

60. What else would you like to share about how you support or have supported the person with cancer in making health and treatment decisions?

[free response]

61. Finally, would you be willing to participate in a follow up survey and/or discussion about your caregiving experience?

[singleSelect - text]

[fixed]

*a. Yes

*b. No

*c. Not Sure

62. You indicated you would be willing to participate in a follow-up survey and/or discussion about your caregiver experience. If so, please provide your name, email address and phone number. <br>

[free response]

63. We realize your time is valuable and are very grateful to you for completing this survey. Your input will help a patient advocacy organization to improve its programs for caregivers of people with cancer.

[free response]

Sample size: 2703

Targeting reqs: Age: 18+ yrs

Country: US
